# A Cross-Cultural Study of Self-Defining Memories in Chinese and American College Students

**DOI:** 10.3389/fpsyg.2020.622527

**Published:** 2021-01-20

**Authors:** Yuening Wang, Jefferson A. Singer

**Affiliations:** Department of Psychology, Connecticut College, New London, CT, United States

**Keywords:** self-defining memories, narrative identity, cross-cultural study, personality traits, memory functions

## Abstract

Self-defining memories (SDMs) are touchstones in individuals’ narrative identity. This is the first SDM study to compare college students from the mainland People’s Republic of China (PRC) to American college students. It examined SDMs, Big Five personality traits, and memory function in 60 students from each country (*n* = 120). Participants rated their memories for affect, recall frequency, and importance. Chinese students recalled their most positively rated memories more frequently and with greater importance, while American students did not show this pattern. American students who scored higher in Openness were more likely to recall negative memories. Memory content coding revealed that Chinese students recalled significantly more guilt/shame events than American students. Further analysis indicated that these memories were particularly focused on academic performance and parental expectations. The discussion suggests that follow-up studies look at differing emotion regulation strategies in the two countries, as well as at how the two different educational systems are affecting late adolescent identity formation processes.

## Introduction

Over the last 25 years, a body of research has emerged on “self-defining memories” (SDMs) as a distinct form of autobiographical memory (e.g., Singer and Salovey, 1993; Blagov and Singer, 2004; Sutin and Robins, 2005; Singer et al., 2013; Holm et al., 2017). SDMs are personal memories that are vivid, affectively intense, and repetitively recalled, linked to other similar memories, and focused on an enduring concern or unresolved conflict (Moffitt and Singer, 1994). They are specific memories within the life script that provide individualized meaning to narrative identity (Singer et al., 2013; Scherman et al., 2017). Individuals return to these memories as touchstones; they are valuable sources of information about what they want or do not want in their lives (Singer, 2019).

Although at least 150 SDM studies across 19 different countries have been conducted,^1^ there have only been two studies that made direct cross-cultural comparisons of SDMs. In an early study of Australian and Asian undergraduate students, Australians provided more individualistic SDMs, while the Asian participants retrieved more relationship SDMs (Jobson and O’Kearney, 2008). Lardi et al. (2010) compared Swiss and American college students and found not only similar agency and communion themes but also more explicit meaning-making in the Swiss students’ SDMs.

There is a valuable and growing body of research that has looked at cross-cultural differences in autobiographical memory with respect to positive and negative events, the centrality of these events to individuals’ life scripts, and the functions that these remembered events serve for the individual (Wang, 2004; Wang and Conway, 2004; Conway et al., 2005; Liao et al., 2015; Scherman et al., 2015a,b, 2017). Some of these studies have indicated cross-cultural differences in participants from the People’s Republic of China (PRC) vs. Western participants; for example, PRC participants were more likely to provide memories that reflected interdependent vs. individual self-construals (Wang and Conway, 2004; Conway et al., 2005). Additionally, Scherman et al. (2015a) found that while individuals, 40 years or older, across cultures tended to rate positive events as more central to their identity, PRC participants gave negative events higher centrality ratings than non-Asian participants. Based on previous research (Heine and Hamamura, 2007), they postulated that this might be due to a tendency for Chinese participants to engage less in self-enhancement and more in self-criticism in identity consolidation.

Regarding the functions of autobiographical memory across cultures, Wang et al. (2015) found that Chinese-American participants used memories more for lesson-learning and directive functions than their European-American counterparts. However, Kulkofsky et al. (2010) reported that European-American mothers, compared to their Chinese counterparts, reported a greater emphasis on social and problem-solving functions in memories shared with their children.

Functional approaches to memory may also vary among individuals with different personality traits, as measured across the Big Five personality dimensions. For example, people high in extraversion share narratives for social reasons (John and Srivastava, 1999; Baddeley and Singer, 2008; Bluck and Alea, 2011). In contrast, the trait of conscientiousness has been shown to be linked to directive functions and to place an emphasis on brief factual narratives based on instrumental reasons (Baddeley and Singer, 2008). Openness to experience has been related to using memory for self and directive functions, while people high in neuroticism frequently recalled memories to establish self-continuity (Rasmussen and Berntsen, 2010).

Studies have also looked at the relationship of the Big Five personality traits to memory functions across cultures (Rasmussen and Habermas, 2011; Harris et al., 2014; Alea et al., 2015; Liao et al., 2015; Maki et al., 2015). Openness has been linked to directive functions in participants from Western, Educated, Industrialized, Rich and Democratic (WEIRD) countries (see Henrich et al., 2010; Alea et al., 2015; Alea and Wang, 2015). Liao et al. (2015) examined Taiwanese and American young adults for memory functions of self-continuity, social-bonding, and directing-behavior. The results showed that, compared to the Americans, Taiwanese young adults who scored higher in conscientiousness used their SDMs more frequently to create self-continuity. The authors suggested that Taiwanese young adults who are seeking to develop more individualized identities require more reliance on their personal memories than American young adults who have an existing social context that encourages individuation.

These cross-cultural examinations of memory affect and function, as well as the memory’s relationship to the Big Five personality traits, suggest a valuable starting point for an exploratory study of SDMs in participants from the United States and the PRC. The studies cited above have ranged from children to young adults, mothers, and participants over 40. We elected to begin with a study of comparable college-aged samples in the two countries. Knowing the marked difference in their respective high school educational systems, we were also curious if this might be reflected in the content of their memories (e.g., Cohen and Spillane, 1992; Wang and Lin, 2005; Li and Ni, 2012; Li et al., 2013; Wang et al., 2015).

Since the essence of SDMs is their ongoing role in the participant’s sense of self and goal structure, participants were asked to rate the memory’s importance to them, the frequency of recall, and how the memory made them feel in the current moment rather than at the original event’s occurrence. A well-established content coding system for SDMs (Thorne and McLean, 2001) allowed for a comparison of major memory themes in the domains of achievement and relationship, among other categories. SDM studies tend to collect multiple memories and require individuals to record narratives of the selected memories, thereby leading to a lengthier administration per participant and smaller sample sizes, which was an inherent limitation of this initial study.

To examine memory function, we employed revised thinking about life experience scale (TALE) of Bluck and Alea (2011), consisting of three subscales: self-continuity, social-bonding, and directing-behavior. To assess the Big Five personality traits, we relied on the 10-item Big Five Inventory (BFI-10), covering the five domains of Openness, Conscientiousness, Extraversion, Agreeableness, and Neuroticism.

Given that this is the first study that directly compares SDMs in Western vs. PRC individuals, we approached any predictions of differences with some trepidation and more in a spirit of exploration. However, based on the above review of the previous studies of more general autobiographical memories in Western and Chinese samples, we proposed the following tentative predictions. First, given the Scherman et al. findings of greater centrality for positive events across cultures, we predicted that both samples would rate positive events as more important in their lives. Second, again following Scherman et al. we proposed that the Chinese students would assign greater importance to their negative memories than the American students. Drawing on the work of Heine and colleagues, we also predicted that the Chinese sample might be less inclined to engage in self-enhancing SDMs and more prone to reflect themes of self-criticism in their SDMs, especially around familial or societal expectations. If, as an earlier research of Jobson and O’Kearney (2008) and Wang suggested, our Chinese sample was to draw on more interdependent self-construals, we expected this group to display more relational themes vs. achievement/autonomy themes in their memory content, relative to the American sample. Finally, we predicted, based on the Liao et al. (2015) and the Wang et al. (2015) findings, respectively, that we might see greater reliance on directive functions and a relationship between conscientiousness and self-continuity in the sample from the PRC.

## Materials and Methods

### Participants

There were 60 Connecticut College students from New London, Connecticut (*M* = 19.53 years, *SD* = 1.38, 48 women) and 60 Chinese students (*M* = 20.15 years, *SD* = 1.18, 44 women) from Beijing Normal University in China. Chinese students received the equivalent of $10 for their participation. American students received either course credit or $10 dollars. All measures were distributed by paper and pencil. Americans reported ethnicity as African American (0%); European American or White (71.7%); Hispanic, Latino, or Latina (18.3%); Asian American (6.7%); and other (3.3%). All Chinese participants were from the mainland PRC.

### Materials

#### Self-Defining Memory Request and Rating Scales

To collect the two SDMs, participants received the SDM request (Singer and Blagov, 2000). They wrote down two SDMs, rated how frequently they thought about them (1 = *less than once a year*, 5 = *more than once a week*), how important they were to them (1 = *not at all important*, 5 = *extremely important*), and, on separate scales, how positively and negatively they felt at current recall (1 = *not at all positive or negative*, 5 = *extremely positive or negative*).

#### Thinking About Life Experience Scale

The TALE is a brief, valid measure of three functions of autobiographical memory (Bluck and Alea, 2011): self-continuity, social-bonding, and directing-behavior (five items per subscale). Sample items (Likert scales − 1 = *almost never*, 5 = *very frequently*) included: *I frequently recall this memory because I want to understand who I am now* (self-continuity); *when I want to develop more intimacy in a relationship* (social-bonding); and *when I want to try to learn from my past mistakes* (directing-behavior). Cronbach’s alphas for self-continuity, social-bonding, and directing-behavior were respectively: 0.77, 0.88, and 0.70 (Chinese) and 0.78, 0.85, and 0.74 (American).

#### The Big Five Inventory

A 10-item version of the BFI (developed to facilitate cross-cultural research; Rammstedt and John, 2007) was used to assess Openness, Conscientiousness, Extraversion, Agreeableness, and Neuroticism. Participants responded using Likert scales (1 = *strongly disagree*, 5 = *strongly agree*) endorsed two items per subscale, e.g., *I see myself as someone who is generally trusting* for Agreeableness.

#### Demographics

Participants reported their age, gender, and race (Chinese participants were not asked for race).

#### Manual for Coding Events in Self-Defining Memories

Thorne and McLean (2001) categorized SDMs into six types of events: life threatening events (LTEs), recreation/exploration, relationship, achievement, guilt/shame, and drug, alcohol, or tobacco use. Each narrative can only be coded into one category. Each memory was scored by the first author and then by a second independent rater. In the beginning, the two raters scored 240 memories for training and achieved a 90 percent inter-rater agreement. Then, the two raters each scored 50% of the actual memories from the study and achieved a 92 percent inter-rater agreement. All the discrepancies were discussed and resolved.

### Procedure

After providing informed consent, participants in both countries filled out the same battery of questionnaires in paper and pencil format in the following order: *SDM Request*, *Memory Rating Scales*, *TALE*, *The BFI-10*, and *Demographics*.

The study was conducted in classrooms in each country and participants received the questionnaires in groups of 5–6. Completion took approximately 30–40 min. For administration in China, all measures were translated into Chinese, back-translated by a translator fluent in both English and Chinese. The original and the back-translated versions were compared for accuracy and any small adjustments were then applied to the translated version. Besides the language of administration, measures and instructions were identical across cultures.

## Results

### Comparison of Importance, Memory Affect, Frequency, and Function for the Two Samples

There were neither significant differences in overall frequency, importance, or memory affect in the two samples nor differences in functions and personality traits (see Tables 1, 2). Both samples rated their memories as quite important in their lives (means between 8.15 and 8.45 out of a possible 10 – indicating very to extremely important) and recalled them frequently (means between 6.18 and 6.67 out a possible 10, which translates to more than once a month per memory). Each sample also rated their average memory affect as more positive than negative across the two memories. This finding did conform to the results of Scherman et al. that, across cultures, memories of personal significance tend to be more positive than negative. Contrary to our prediction, the Chinese sample neither did show a tendency to rate their negative memories more highly than their American counterparts nor did it show a higher rate of directive functions for their memories.

**Table 1:** Self-Defining Memories’ (SDMs) Ratings for Chinese vs. American College Students.

	Chinese		American	
	M	SD	M	SD
Frequency	6.67	1.28	6.18	1.68
Importance	8.15	1.48	8.45	1.36
PA	7.10	2.21	6.50	1.99
NA	4.78	2.01	5.08	1.90

*N*=60 for each group. Ranged 2–10 for two memories combined. PA, positive affect; NA, negative affect. MANOVAs, *F*(4,115) = 2.86, Wilks’ Lambda = 0.91, *p* = n.s.

**Table 2:** Descriptive statistics of memory functions and personality traits for Chinese vs. American College Students.

	Chinese		American	
	M	SD	M	SD
Self	30.88	7.04	29.18	7.83
Social	26.80	7.41	25.42	7.98
Directive	30.27	7.68	31.27	7.81
Extraversion Agreeableness Conscientiousness Openness Neuroticism	5.82 7.03 5.67 6.93 6.15	2.27 1.50 1.61 1.71 1.91	6.07 6.88 7.67 7.73 6.77	2.28 2.19 1.66 2.12 2.00

*N* = 60 for each group. Scores of functions ranged from 10 to 50 for five items per subscale in each memory added up. Scores of personality traits ranged from 2 to 10 for the two items per subscales combined.

Both the American and Chinese sample showed a strong positive correlation between importance and frequency of SDM recall, *r* = 0.53, *p* < 0.01, *r* = 0.42, *p* < 0.01, respectively (see Table 3). A major difference between the two countries in the intercorrelations of the memory variables was that, for Chinese students, the more positively the memory was rated, the more frequently it was recalled, *r* = 0.48, *p* < 0.05, and the more important it was rated, *r* = 0.57, *p* < 0.05 (see Table 3). These two relationships did not emerge in the American sample.

**Table 3:** Intercorrelations among Memory Variables for Chinese vs. American College Students.

	Frequency		Importance		PA		NA	
	C	A	C	A	C	A	C	A
Frequency	1	1	0.53^**^	0.42^**^	0.48^*^	0.03	0.11	0.13
Importance			1	1	0.57^*^	0.09	0.20	0.06
PA					1	1	−0.76	−0.62
NA							1	1

*N* = 60 for each group. PA, positive affect; NA, negative affect. C represents Chinese Sample; A represents American Sample.

* *p* < 0.05

** *p* < 0.01.

Next, we looked at the relationship of the memory variables to the three functions as measured by the *TALE* within each sample. Both Chinese and American students reported that memories that were used for directive and goal-pursuit functions were more frequently recalled, *r* = 0.30, *p* < 0.05; *r* = 0.32, *p* < 0.05, respectively, and rated as more important, *r* = 0.27, *p* < 0.05; *r* = 0.46, *p* < 0.01, respectively.

### Relationships Among SDM Variables and Functions and Personality Traits in the Two Samples

There were significant correlations between the Big Five personality traits and the SDM ratings of importance, frequency, and memory affect (see Table 4). In the Chinese sample, there was a positive correlation between extraversion and frequency of SDM recall, *r* = 0.41, *p* < 0.01. In contrast, American students displayed a significant positive relationship between openness and negative memory affect, *r* = 0.33, *p* < 0.05, and a negative relationship between openness and positive memory affect, *r* = −0.28, *p* < 0.05. Regarding our prediction of a relationship between conscientiousness and self-continuity in the Chinese sample, there was no significant relationship (*r* = −0.14, *ns*).

**Table 4:** Correlations between Personality Traits and SDM Ratings.

	Extraversion		Agreeableness		Conscientiousness		Openness		Neuroticism	
	C	A	C	A	C	A	C	A	C	A
Frequency	0.41^**^	0.01	0.16	−0.02	0.14	0.07	−0.02	−0.17	−0.07	−0.03
Importance	0.025^*^	−0.02	−0.06	0.10	0.21	0.28^*^	−0.07	0.03	0.04	−0.14
PA	0.14	0.18	−0.06	0.28^*^	0.31^**^	0.14	−0.03	−0.28^*^	−0.12	−0.18
NA	0.07	−0.09	0.00	−0.25	−0.24	−0.03	−0.08	0.33^*^	0.04	0.17

*N* = 60 for each group. PA, positive affect; NA, negative affect. C represents Chinese Sample; A represents American Sample.

* *p* < 0.05

** *p* < 0.01.

### Differences in Memory Content and Themes in the Two Samples

Although the Chinese sample showed a higher percentage of relationship memories (34%) compared to the American sample (24%), a *χ* ^2^(1) = 0.202 was non-significant, indicating no reliable difference in interdependent self-construal between the two samples. However, there was one significant difference that was found for guilt/shame themes between the samples. A significant association was found between Country and guilt/shame memories, *χ* ^2^(1) = 5.82, *p* < 0.05, with Chinese students (18.3%) reporting more guilt/shame memories than Americans (10%). After reviewing the memories, it was noted that there were striking differences in the mention of academic stress in relation to high school experience. Accordingly, two independent raters rated the memories for the presence of academic stress (e.g., any mention of worry or anxiety regarding examinations, teachers, evaluations, parental pressure, and workload). Of 120 Chinese SDMs, 28 (23.3%) were related to academic stress, compared to four (3.33%) in the American sample. Among Chinese students’ guilt/shame memories, 45.5% of them were relevant to academic stress, while none of the guilt/shame SDMs in the American sample were related to academics. These findings of more negative self-evaluation with regard to academics did provide support for Heine’s proposal that there might be more inclination to self-criticism in an East Asian sample, especially in areas related to familial or social expectations.

## Discussion

The current study examined the affect, function, and content of SDMs and their relationship to personality in emerging adults in the PRC and the United States. As the first study to examine SDMs in a sample of college students from the PRC, it has demonstrated that, similar to an American college student sample, these students rate their SDMs as very to extremely important, as having strong affective intensity, and as being recalled with impressive frequency.

Additionally, both Chinese and American results confirmed that the greater importance participants assigned to their memories, the more frequently they recalled their memories.

The one difference in memory affect for the two samples was that for Chinese college students, memories with greater positive affect were recalled more frequently and rated more important, while positive affect was not linked to either frequency or importance in the American sample. This result differs from the Scherman et al. (2015a) findings that indicated no difference in their Chinese sample from other countries in the centrality of positive events within their life scripts. Also, in contrast to the findings of Scherman et al. (2015a), the Chinese sample did not show a higher rate of negative memories or attribute greater importance to them compared to the American sample.

Yet, despite apparent greater emphasis on positive memories in this sample of Chinese college students, there was one domain in which they expressed more negative themes than their American counterparts. The Chinese sample reported more guilt/shame memories, specifically in the context of their academic identity. In line with the work of Heine and Hamamura (2007), these memories often reflected self-criticism in light of familial and teachers’ expectations. The students highlighted memories with themes of exam-related stress and academic disappointment. Within these memories, “Gaokao,” the annual Chinese college entrance exam, was the word mentioned most frequently. Students expressed anxiety about poor grades and worry about parental disapproval. One student recalled an experience that she described as common among adolescents faced with the college entrance exam, *“I was very depressed and wanted to escape everything, including my own grades, my confused future, my parents’ scolding, and the look in their eyes. Personally, I’m sensitive. I just want to hide.”* Future SDM studies should explore how this powerful factor in the Chinese educational system may be affecting identity formation processes in Chinese adolescents and young adults (see also, Chao, 2001).

Turning to the memory functions, contrary to our prediction, the Chinese sample did not show a preference for the directive function over the other two memory functions. Individuals in both countries showed roughly equal use of all three functions: self-continuity, social-bonding, and directing-behavior. However, in both samples, more frequently recalled and important memories were significantly linked to the directive function. These findings highlight the role that SDMs with their strong relationship to ongoing goals play in individuals’ lesson-learning and problem-solving (Moffitt and Singer, 1994; Singer et al., 2013).

Unlike the Liao et al. (2015) study, no relationship between conscientiousness and self-continuity emerged. One possible explanation for this is that the Taiwanese sample, who is seeking to forge a more Westernized identity, may be more reliant on the self-continuity function of memory to build this identity. Participants from the PRC may be less inclined to assert this function in the context of their adherence to a more collectivist culture.

The one striking difference in the relationship of SDMs to personality traits in the two groups was that American students who were higher in openness reported less positive and more negative affective responses to their SDMs. This finding may suggest a willingness in these individuals to reflect on more negative experiences for potential meaning-making. This tendency in this sub-group within the American sample contrasted with the Chinese students’ overall tendency to think more about their positive memories and rate them as more important.

This initial study of SDMs in the PRC suffers from several limitations. It utilized a small sample and was focused on a narrow age band of college students; it was also skewed to female participants and based in an urban center. In general, SDM studies elicit more than two memories, which allows for patterns across the memories to emerge and greater reliability to be achieved. For these reasons, the findings reported should be considered preliminary and of limited generalizability at this stage. However, they have demonstrated the viability of extending SDM research to the PRC and ideally will pave the way for further research across age groups, as well as to examine other SDM variables, such as memory specificity or distribution across the lifespan.

## Conclusion

This investigation extended the study of SDMs, a unique form of autobiographical memory, to a sample of college students from the PRC. It demonstrated that similar to the American college student sample, participants related their SDMs as highly important and frequently recalled. In contrast to the American sample, Chinese students tended to rate more positive memories as more important and recalled them more frequently than less positively rated memories. Both samples used their SDMs as guides for directing their behavior and as a method of articulating their goals. When examining personality factors, American students linked openness to a tendency to recall more negative SDMs. With regard to memory content, Chinese college students recalled more SDMs related to academic stress and expressed themes of shame/guilt in relation to these memories; American college students did not show this pattern. These findings suggest the value of further cross-cultural comparisons that would look more closely at both patterns of affect management and self-enhancement/criticism and effects of academic socialization and stress, as reflected in narrative identity.

## Data Availability Statement

The raw data supporting the conclusions of this article will be made available by the authors, without undue reservation, to any qualified researcher.

## Ethics Statement

The studies involving human participants were reviewed and approved by The Institutional Review Board of the Department of Psychology of Connecticut College. The patients/participants provided their written informed consent to participate in this study.

## Author Contributions

YW: conceptualization, project administration, writing, and statistical analyses. JS: conceptualization, supervision, review, writing, and editing. Both the authors contributed to the article and approved the submitted version.

### Conflict of Interest

The authors declare that the research was conducted in the absence of any commercial or financial relationships that could be construed as a potential conflict of interest.

## Footnotes

^1^http://www.self-definingmemories.com/
